# Human leukocyte antigen F-associated transcript 10 regulates the *I_Ks_* potassium channel by competing for Kv7.1 ubiquitination

**DOI:** 10.1098/rstb.2022.0167

**Published:** 2023-06-19

**Authors:** Chen Chen, Xin Zhu, Jinyan Xie, Xiaoqing Li, Rong Wan, Kui Hong

**Affiliations:** ^1^ Department of Cardiovascular Medicine, The Second Affiliated Hospital of Nanchang University, Nanchang of Jiangxi 330006, People's Republic of China; ^2^ Department of Genetic Medicine, The Second Affiliated Hospital of Nanchang University, Nanchang of Jiangxi 330006, People's Republic of China; ^3^ Jiangxi Key Laboratory of Molecular Medicine, Nanchang of Jiangxi 330006, People's Republic of China

**Keywords:** FAT10, *I_Ks_*, Kv7.1, ubiquitination, hiPSC-CMs

## Abstract

The protein expression and function changes from the slow-delayed rectifying *K*^+^ current, *I_Ks_*, are tightly associated with ventricular cardiac arrhythmias. Human leukocyte antigen F-associated transcript 10 (FAT10), a member of the ubiquitin-like-modifier family, exerts a protective effect against myocardial ischaemia. However, whether or how FAT10 influences the function of *I_Ks_* remains unclear. Here, human-induced pluripotent stem cell-derived cardiomyocytes (hiPSC-CMs) and *Fat10* knockout HEK293 (*Fat10^−/−^*) cells through CRISPR-Cas9 technology were used to evaluate the novel modulation of FAT10 in *I_Ks_* function. Patch-clamp studies showed that the overexpression of FAT10 significantly enhanced the current density of *I_Ks_* both in hiPSC-CMs and HEK293-*Fat10^−/−^* cells. In addition, a shortened action potential duration (APD) was seen from hiPSC-CMs transfected with the *ad-Fat10* virus. Then, a series of molecular approaches from neonatal rat cardiomyocytes, H9C2 cells and HEK293 cells were used to determine the regulatory mechanism of FAT10 in *I_Ks_*. First, western blot assays indicated that the expression of Kv7.1, the alpha-subunit of *I_Ks_*, was increased when FAT10 was overexpressed. Furthermore, immunofluorescence and co-immunoprecipitation assays demonstrated that FAT10 could interact with Kv7.1. Notably, FAT10 impedes Kv7.1 ubiquitination and degradation, thereby stabilizing its expression. Finally, a hypoxia model of hiPSC-CMs was established, and the overexpression of FAT10 showed a protective effect against hypoxia-induced decreases in the current density of *I_Ks_*. Taken together, these findings revealed a novel role of FAT10 in the regulation of the *I_Ks_* potassium channel by competing for Kv7.1 ubiquitination, which provides a new electrophysiological insight that FAT10 could modulate Kv7.1.

This article is part of the theme issue ‘The heartbeat: its molecular basis and physiological mechanisms’.

## 1. Introduction

Normal heart rhythm requires the finely orchestrated activity of cardiac ion channels, such as *K*^+^ channels, Na^+^ channels and Ca^2+^ channels, and balance disruption could lead to severe cardiac arrhythmias [1]. To date, the therapeutic effect of antiarrhythmic drugs is unclear because the molecular mechanism of arrhythmia is a mystery. To reveal the pathogenesis of arrhythmia, discoveries of new targets are the key points in antiarrhythmic treatment.

The alpha-subunit Kv7.1, encoded by the *KCNQ1* gene, coassembles with the beta-subunit mink KCNE1 (also known as mink) to generate the slow component of the delayed rectifier *K*^+^ current *I_Ks_*, which is necessary to maintain normal heart rate and rhythm. Loss- or gain-of-function of *KCNQ1* results from abnormal changes in the expression of Kv7.1 and reduced or increased changes in *I_Ks_* currents, ultimately leading to various cardiac arrhythmias [2], which demonstrates that the expression level of Kv7.1 in cardiomyocytes is a critical condition for its function. Within the past few years, Kv7.1 has been known to undergo neddylation by Nedd4-2, a ubiquitin E3 ligase, and subsequent degradation by the ubiquitin–proteasome system (UPS). In addition, Kv7.1 is modified by the small ubiquitin-like modifier (SUMO) in a manner that depends on KCNE1 accessory subunits, leading to stepwise depolarizing shifts in the activation voltage [3]. Therefore, the post-translational modification of Kv7.1 is critical for regulating cardiac *K*^+^ channels.

Cardiac ischaemia can change the outward currents responsible for repolarization. For instance, when cardiac ischaemia is induced, the contribution of adenosine triphosphate-sensitive potassium current (*I_KATP_*) to repolarization increases in the ischaemia heart, while rapid delayed rectifier *K*^+^ current (*I_Kr_*) and inwardly rectifier *K*^+^ current (*I_K1_*) are lessened in comparison, which ultimately leads to a shortening of the action potential duration (APD) [4]. Furthermore, studies have proposed that chronic ischaemia results in decreased *I_Ks_* current density and downregulated *KCNQ1* mRNA expression [5,6]. Thus, the stable maintenance of *I_Ks_* currents during cardiac ischaemia is essential to prevent ventricular arrhythmias.

Ubiquitin-like proteins (UBLs), a family of small proteins with structural and functional similarity to ubiquitin [7,8], are known to modulate ion channels. For instance, SUMO targets the current density of Cav1.2, which is associated with cardiomyopathy and sudden death [9]. SUMO also modifies potassium Kv4.2, thereby increasing surface expression and decreasing current amplitude [10]. Human HLA-F adjacent transcript 10 (FAT10) is a UBL that could stabilize its substrates by antagonizing ubiquitination, as reported by our group [11–13]. We also identified that FAT10 could protect against ischaemia ventricular arrhythmia by binding to Nav1.5 [14]. However, whether FAT10 affects *I_Ks_* currents under physiological or ischaemic/hypoxic conditions has not been established. Here, after the application of human-induced pluripotent stem cell-derived cardiomyocytes (hiPSC-CMs) and a *Fat10* knockout HEK293 (*Fat10^−/−^*) cell model, we demonstrate the novel function of FAT10 in the regulation of the *I_Ks_* channel. FAT10 enhanced the currents of *I_Ks_* under physiological conditions. In addition, FAT10 was related to ameliorating the hypoxia-induced reduction in *I_Ks_* currents. Mechanistically, FAT10 enhanced *I_Ks_* currents by modulating Kv7.1 degradation. Notably, FAT10 competed with the ubiquitination of Kv7.1. Our results indicate that FAT10 could be a potential therapeutic target for arrhythmia in the future.

## 2. Material and methods

### (a) Plasmids and reagents

Plasmids and reagents are described in the electronic supplementary material and methods.

### (b) Culture of human-induced pluripotent stem cell-derived cardiomyocytes

HiPSC-derived cardiomyocytes were purchased from Help Regenerative Medicine Technology Co., Ltd (Nanjing, China) and cultured in Minimum Essential Medium Eagle-alpha modification (Gibco^®^, Gaithersburg, MD, USA) containing 10% fetal bovine serum (FBS).

### (c) Patch-clamp recordings of human-induced pluripotent stem cells

Patch-clamp experiments were carried out using an Axopatch 200B amplifier (Axon) as previously described. For *I_Ks_* recording, hiPSC-CMs were recorded with an intracellular fluid: 125 mM K-aspartic, 20 mM KCl, 1 mM MgCl_2_, 5 mM EGTA, 5 mM HEPES, 2 mM Na-GTP, 5 mM Mg-ATP, 2 mM Na-phosphocreatine, 10 mM EGTA. The external bath solution contained: 150 mM NaCl, 5.4 mM KCl, 1 mM MgCl_2_, 1.8 mM CaCl_2_, 15 mM glucose, 15 mM HEPES, 1 mM Na-pyruvate, 0.2 mM CdCl_2_ and 1 mM BaCl_2_. The action potential recordings of hiPSC-CMs are described in the electronic supplementary material and methods.

### (d) Patch-clamp recordings of transfected HEK293 cells

The *I_Ks_* current of HEK293 cells was recorded using a previously described method [15]. Briefly, the pipette solution contained 45 mmol l^−1^ KCl, 100 mmol l^−1^ K-aspartate, 5 mmol l^−1^ ATP-Mg, 1 mmol l^−1^ CaCl2, 5 mmol l^−1^ EGTA, 5 mmol l^−1^ HEPES and 5 mmol l^−1^ phosphocreatine-disodium (pH adjusted to 7.2 with KOH). The extracellular solution contained 0.33 mmol l^−1^ NaH_2_PO_4_, 140 mmol l^−1^ NaCl, 5.4 mmol l^−1^ KCl, 1.8 mmol l^−1^ CaCl_2_, 0.5 mmol l^−1^ MgCl_2_, 5.5 mmol l^−1^ glucose and 5.0 mmol l^−1^ HEPES (pH adjusted to 7.4 with NaOH). Pipette resistance ranged from 3 to 5 MΩ. Slow potassium (I_Ks_) currents were obtained by depolarizing voltage steps from a holding potential of 80 mV to various test potentials. Fitmaster was used to collect data. The current–voltage (I–V) relation was fit by the Boltzmann equation.

### (e) Isolation and culture of neonatal rat cardiomyocytes

Neonatal rat cardiomyocytes (NRCMs) were isolated from the ventricular myocardia of newborn SD rats through an enzymatic digestion method according to previous studies [16,17]. In brief, hearts were removed aseptically and minced into small fragments, then digested with trypsin at 37°C under agitation. Then trypsin was removed by centrifugation at 300*g* for 5 min. After repeats of digestion, isolated cells were plated into the dish for 2 h in Dulbecco's modified Eagle's medium (DMEM) with 5% fetal calf serum in a 37°C incubator, and 5% CO_2_. Then non-attached NRCMs were collected and incubated under the same conditions as above.

### (f) Cell culture and transfection

HEK-293 and H9C2 cell lines were purchased from ATCC (Rockville, USA). Cells were grown in DMEM (Thermo Fisher Scientific) supplemented with 10% FBS (Biochrome, Berlin, Germany), 100 u ml^–1^ penicillin and 100 µg ml^−1^ streptomycin. All cells were kept in a 37°C incubator containing 5% CO_2_.

Transfection of plasmid DNA and siRNA was carried out using Lipofectamine^i^™ 3000 (Invitrogen, USA) according to the manufacturer's instructions. Cells were seeded in six-well plates overnight, and transfection was carried out when cells approached 80% confluence.

In addition, the experiment of hiPSC-CMs in response to hypoxia was as follows. The cells were pre-treated with *ad-Fat10* or *ad-con* for 48 h in DMEM and subsequently subjected to hypoxia for 4 h.

### (g) Generation of CRISPR-Cas9 knockout of the *Fat10* gene in HEK293 cell lines

The generation of CRISPR-Cas9 knockout of the *Fat10* gene in HEK293 is referred to a previous report [14]. HEK293 cells were transfected with CRISPR-Cas9 plasmids for 2 days and then treated with puromycin to enrich transfected cells. The surviving cells were sorted into single clones on the 96-well plate. Then 80% of cells per well were used for DNA isolation and genotyping after one week. The remaining 20% of cells were cultured for 8 to 10 days, and clones propagated from single cells were picked out. *Fat10* knockout HEK293 cells were confirmed by both WB and DNA sequencing.

### (h) Western blot analysis and quantitative real-time polymerase chain reaction

Western blot and quantitative real-time polymerase chain reaction (qRT-PCR) were performed using standard methods [13,17]. For western blot, cells were lysed in RIPA lysis buffer. Protein concentrations were quantified using the BCA Protein Assay Kit. Cell proteins were separated via 6–15% SDS-PAGE and transferred to 0.45 µm thick polyvinylidene difluoride membranes. After blocking the blotted membranes with 5% skim milk for 1 h at room temperature, membranes were incubated with primary antibodies (1 : 1000) overnight at 4°C and then with secondary antibodies (1 : 2000) for 1 h at room temperature. All the antibodies used in this study are shown in the electronic supplementary material.

Total RNA in NRCMs was extracted using TRIzol (TaKaRa, Japan) and then reverse transcribed into cDNA. All procedures were conducted using TB Green^®^ Premix Ex Taq^TM^ following the manufacturer's protocols. Quantification was performed using the 2-ΔΔCT method after normalization of the expression levels against GAPDH. The primer sequences are listed in the electronic supplementary material.

### (i) Co-immunoprecipitation assay

Total proteins were extracted from cells using RIPA buffer. Five hundred micrograms of protein lysates, combined antibody, was incubated for 1–2 h at room temperature with mixing. Then, 25 µl Protein A/G magnetic beads were added and the mixture was incubated at 4°C overnight with mixing. The obtained immune complexes were washed three times, boiled with 2× SDS-PAGE Sample Loading Buffer, and subsequently detected by western blot.

### (j) Immunofluorescence staining

NRCMs were fixed with methanol for 30 min and then cells were treated with 0.3% Triton X-100 for 30 min at room temperature. Then, cells were blocked with 5% goat serum for 30 min at room temperature. Afterward, the primary antibody diluted in FBS was added to cells for incubation at 4°C overnight. On the second day, cells were incubated with DyLight 488 nm goat anti-mouse IgG (H + L) for 90 min at room temperature, and cell nuclei were stained with DAPI. Finally, cells were viewed under a confocal microscope.

### (k) Statistical analysis

Statistical analysis was performed using GraphPad Prism version 8.0. The results were expressed as mean ± s.e.m. for experiments conducted at least in triplicates. An unpaired *t*-test was used for comparison between two groups, ANOVA was used for three or more groups, and *p*-value less than 0.05 was considered to be significantly different.

## 3. Results

### (a) FAT10 enhances the currents of *I_Ks_* and shortens the action potential duration in both human-induced pluripotent stem cells and *Fat10^−/−^*-HEK293 cells

**Figure 1.**
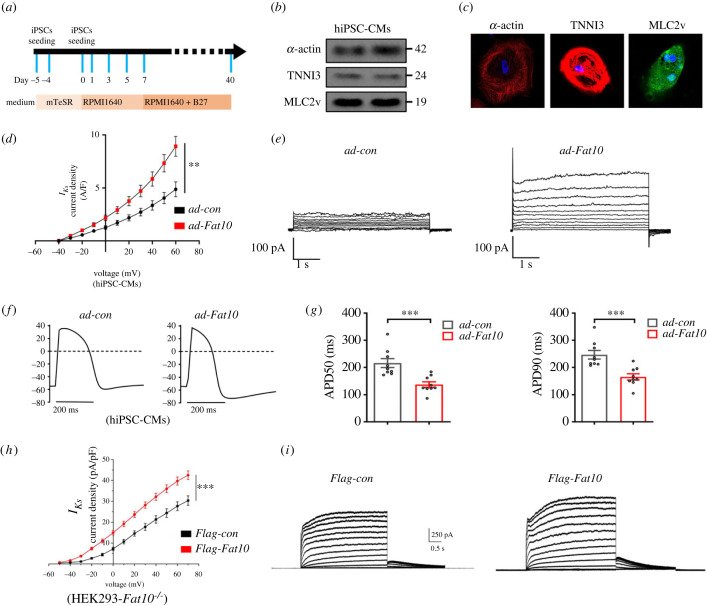
Effects of FAT10 overexpression on *I_Ks_* current densities. (*a*) Schematic diagram of the generation of hiPSC-CMs generated. (*b*) Representative immunoblots of TNNI3, α-actin and MLC2v expression in hiPSC-CMs as detected by WB. (*c*) Representative immunofluorescence assays of hiPSC-CMs expressing α-actin (left, red), TNNI3 (middle, red) and MLC2v (right, green). (*d*) Mean current density versus voltage relationships for hiPSC-CMs transfected with *ad-Fat10* (*n* = 15) or *ad-con* (*n* = 12). (*e*) Representative current traces were recorded in hiPSC-CMs transfected with *ad-Fat10* or *ad-con*. (*f*) Representative APs were recorded in hiPSC-CMs when transfected with *ad-Fat10* (right) (*n* = 9) or *ad-con* (left) (*n* = 9). (*g*) The duration of APD_50_ and APD_90_ in hiPSC-CMs when transfected with *ad-Fat10* or *ad-con*. (*h*) Mean current density versus voltage relationships for HEK293T-*Fat10^−/−^* cells. (*i*) Representative current traces recorded in HEK293T-*Fat10^−/−^* cells expressing KCNQ1 with KCNE1. *p < 0.05, **p < 0.01, ***p < 0.001 and n.s. = not significant.

*I_Ks_* currents serve as a repolarizing current of ventricular cardiomyocytes mainly in humans and various mammals. To explore the functional role of FAT10 in *I_Ks_* under physiological conditions in human cardiomyocytes, hiPSC-CMs were generated (figure 1*a*). Our western blot (figure 1*b*) and immunofluorescence analysis (figure 1*c*) demonstrated that hiPSC-CMs exhibited positive staining for the regular arrangement of sarcomeric proteins such as TNNI3, *α*-actin and MLC2v. Then, we treated hiPSC-CMs with the *ad-Fat10* virus to overexpress FAT10 (over-FAT10), and whole-cell *I_Ks_* currents were recorded by patch-clamp technique. As shown in figure 1*d*,*e*, over-FAT10 significantly enhanced the current density of *I_Ks_* when compared to that in the control group. *I_Ks_* are one of the major contributors to repolarization currents that modulate ventricular APD. Therefore, we further observed whether FAT10 could alter APD. Our results showed that over-FAT10 significantly shortened APD (APD50: *p* = 0.0009; APD90: *p* = 0.0007) (figure 1*f*,*g*). Then, to corroborate the above findings, a heterologous expression system was used to generate *Fat10* knockout HEK293 (*Fat10^−/−^*-HEK293) cells by transfection with *CRISPR-Cas9* plasmids [12]. We performed whole-cell recordings in *Fat10^−/−^*-HEK293 cells cotransfected with *KCNQ1* and *KCNE1* alone or in combination with FAT10. Similar to the results in hiPSC-CMs, the *I_Ks_* current was significantly enhanced when cells were transfected with *Flag-Fat10* plasmid (figure 1*h*,*i*).

### (b) FAT10 increases the expression of Kv7.1 protein

**Figure 2.**
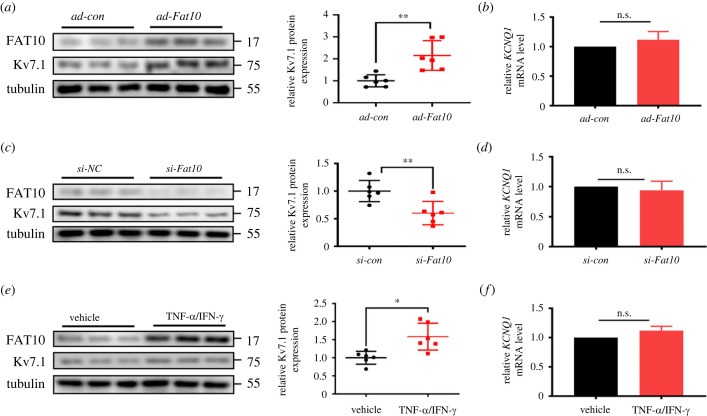
FAT10 regulates Kv7.1 protein expression in cardiomyocytes. (*a*) Representative immunoblots (left) and quantitative assessment of the protein (right) levels of Kv7.1 in NRCMs treated with *ad-Fat10.* (*b*) Kv7.1 mRNA level as measured by qRT-PCR in cultured NRCMs after treatment with *ad-Fat10*. (*c*) Representative immunoblots (left) and quantitative assessment of the protein (right) levels of Kv7.1 in NRCMs treated with *si-Fat10*. (*d*) Kv7.1 mRNA levels were measured by qRT-PCR in cultured NRCMs after treatment with *si-Fat10*. (*e*) Representative immunoblots (left) and quantitative assessment of the protein (right) levels of Kv7.1 in NRCMs treated with TNF-α/IFN-γ. (*f*) Kv7.1 mRNA levels were measured by qRT-PCR in cultured NRCMs after treatment with TNF-α/IFN-γ. *p < 0.05, **p < 0.01, ***p < 0.001 and n.s. = not significant. The data shown are the mean of three independent experiments.

Next, we unravelled the effective mechanism of FAT10 on *I_Ks_*. *I*_Ks_ are conducted by the pore-forming *α*-subunit Kv7.1 in a complex with the β-subunit KCNE1(also known as mink). The results showed whether upregulation or downregulation of FAT10 can affect the expression of Kv7.1 but not KCNE1 protein (electronic supplementary material, figure S3), which demonstrates that FAT10 mediates the *I_Ks_* channel via specific regulation of Kv7.1. In addition, as shown in figure 2*a*, over-FAT10 was associated with increased levels of Kv7.1 protein expression, and down-FAT10 had the opposite effect (figure 2*c*). Moreover, increased Kv7.1 expression was also confirmed by upregulated endogenous over-FAT10 induced by interferon-*γ*/tumour necrosis factor-*α* (IFN-*γ*/TNF-*α*) (figure 2*e*), but no changes in the mRNA levels were observed (figure 2*b*,*d*,*f*). Collectively, these data provide direct evidence that FAT10 regulates the *I*_Ks_ channel by increasing Kv7.1 expression.

### (c) FAT10 stabilizes Kv7.1 expression by inhibiting ubiquitin-mediated degradation

**Figure 3.**
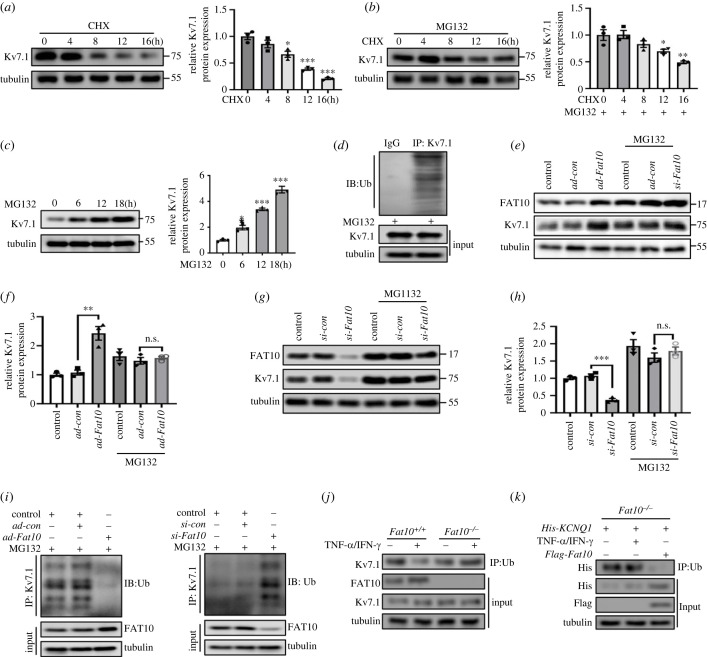
FAT10 inhibits Kv7.1 ubiquitination. (*a,b*) Representative immunoblots (left) and quantitative assessment of the protein (right) levels of Kv7.1 in NRCMs treated with CHX (20 µM) or MG132 (15 µM) for the indicated times. (*c*) Representative immunoblots (left) and quantitative assessment of the protein (right) levels of Kv7.1 in NRCMs treated with MG132 (15 µM) for the indicated times. (*d*) Co-IP between endogenous ubiquitin and FAT10. (*e–h*) Representative immunoblots or quantitative data of Kv7.1 expression in NRCMs after adenoviral overexpression (*e,f*) or knockdown (*g,h*) of *Fat10*. Cells were treated with or without MG132. (*i*) Cardiomyocytes were transfected with the indicated adenovirus constructs. The cells in each group were treated with MG132. Kv7.1 ubiquitination was detected. (*j*) Representative immunoblots of the Kv7.1 ubiquitination levels in *Fat10^+/+^* and *Fat10^−/−^* HEK293 cells treated with or without TNF-*α*/IFN-*γ*. (*k*) Representative immunoblots of the Kv7.1 ubiquitination levels in the indicated groups of *Fat10*^−/−^ HEK293 cells. *p < 0.05, **p < 0.01, ***p < 0.001 and n.s. = not significant. The data shown are the mean of three independent experiments.

To explore the specific regulatory mechanism of FAT10 in Kv7.1 expression in NRCMs, we examined whether Kv7.1 is degraded by the UPS. First, we detected Kv7.1 expression in NRCMs treated with cycloheximide (CHX) in the absence or presence of the proteasome inhibitor MG132. As shown in (figure 3*a*), a significant decrease in endogenous Kv7.1 after CHX treatment was observed in NRCMs following different times (figure 3*a*), but this decrease was not observed in NRCMs when both treated with CHX and the proteasome inhibitor MG132 (figure 3*b*). Besides, cumulative Kv7.1 levels increased as the duration of treatment with MG132 alone increased (figure 3*c*). In addition, we also identified the interaction between FAT10 and ubiquitin by co-immunoprecipitation (Co-IP) (figure 3*d*). Subsequently, the increased Kv7.1 expression upon over-FAT10 and decreased Kv7.1 expression upon down-FAT10 were abolished when NRCMs were treated with MG132 (figure 3*e–h*). Then, the Co-IP results revealed reduced or increased levels of Kv7.1 ubiquitination with over-FAT10 or down-FAT10 in cardiomyocytes, respectively (figure 3*i*). Finally, the reduced binding of Kv7.1 to ubiquitin in *Fat10*^+/+^-HEK293 cells after treatment with IFN-*γ*/TNF-*α* was observed, but not in *Fat10^−/−^*-HEK293 cells (figure 3*j*). Similarly, in the *Fat10^−/−^*-HEK293 cells, the binding of Kv7.1 to ubiquitin was rescued upon expression of *Flag-Fat10*, but not upon treatment with IFN-*γ*/TNF-*α* (figure 3*k*). Taken together, these experiments demonstrated that FAT10 stabilized Kv7.1 expression in cardiomyocytes by inhibiting ubiquitination.

### (d) FAT10 decreases Kv7.1 ubiquitination by competing with ubiquitin binding

**Figure 4.**
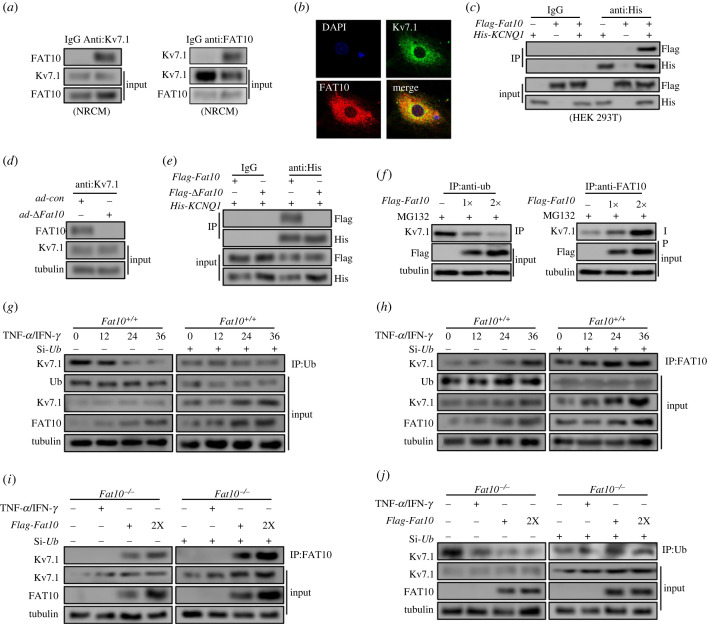
FAT10 stabilizes Kv7.1 by competing with ubiquitin for binding. (*a*) Co-IP for Kv7.1 and FAT10 in cultured cardiomyocytes. (*b*) Colocalization of FAT10 and Kv7.1 in cardiomyocyte cultures; FAT10(1:50) is shown in red, Kv7.1(1:200) is shown in green, and DAPI nuclear counterstaining is shown in blue. (*c*) The association between FAT10 and Kv7.1 in HEK293 cells expressing *Flag-FAT10* and *His-KCNQ1* was detected by Co-IP. (*d*) NRCMs were transfected with *ad-*Δ*Fat10.* Co-IP assays were performed to analyse the binding of FAT10 with Kv7.1. (*e*) The association between Flag and His in HEK293 cells expressing *Flag-*Δ*FAT10* and *His-KCNQ1* was detected by Co-IP. (*f*) H9C2 cells were transfected with increasing amounts of *Flag-Fat10* plasmid. The cells were lysed for immunoprecipitation using anti-Ub and anti-FAT10 beads to detect Kv7.1 binding. (*g,h*) *Fat10^+/+^*HEK293 cells were transfected with *Si-Ub* or *Si-scramble* and treated with IFN-*γ*/TNF-*α* for the indicated times. Co-IP was performed to detect Ub–Kv7.1 and the FAT10–Kv7.1 complex. (*i,j*) *Fat10^−/−^*HEK293 cells were transfected with *Si-Ub*/*Si-con* and the *Flag-Fat10* plasmid. Co-IP was performed to detect Ub–Kv7.1 and FAT10–Kv7.1 complex formation. HEK293 cells were treated with IFN-*γ*/TNF-*α* to induce FAT10 expression. *p < 0.05, **p < 0.01, ***p < 0.001 and n.s. = not significant. The data shown are the mean of three independent experiments.

We previously found that FAT10 stabilized substrates by competitively binding with ubiquitin [13], ultimately reducing substrate ubiquitination. Therefore, we hypothesized that FAT10 could inhibit Kv7.1 ubiquitination by competing with ubiquitin to bind Kv7.1. To prove our conjecture, we first determined whether FAT10 and Kv7.1 could interact. As shown in figure 4*a*, the Co-IP results showed that endogenous Kv7.1 and FAT10 were combined in NRCMs. Colocalization of FAT10 and Kv7.1 was also observed by using confocal assays (figure 4*b*). In addition, the interaction was further confirmed in HEK293 cells transfected with *Flag-Fat10* and *His-KCNQ1* plasmids (figure 4*c*). Furthermore, since FAT10 regulates substrates through its two C-terminal glycine residues, to further demonstrate that FAT10 could bind to Kv7.1, we generated FAT10 mutant adenoviruses lacking two C-terminal glycine residues (*ad*-Δ*Fat10*). As expected, the interactions between FAT10 and Kv7.1 disappeared when cells were transfected with *ad*-Δ*Fat10* (figure 4*d*,*e*).

Next, we analysed whether FAT10 competes with ubiquitin for binding Kv7.1. H9C2 cells were transfected with increasing amounts of *Flag-Fat10*, and immunoprecipitation with anti-ubiquitin and anti-FAT10 beads was then performed applied to detect the binding of Kv7.1. Intriguingly, Co-IP and WB experiments demonstrated that the FAT10–Kv7.1 complex level was gradually increased, while the ubiquitin–Kv7.1 (Ub–Kv7.1) complex level gradually decreased when FAT10 was overexpressed (figure 4*f*). Moreover, endogenous over-FAT10 induced by TNF-*α*/IFN-*γ* increased Kv7.1 expression and FAT10–Kv7.1 complex levels but decrease Ub–Kv7.1 complex levels in a dose-dependent manner (figure 4*g*,*h*). Furthermore, in HEK293-*Fat10^−/−^* cells, when FAT10 expression was rescued by transfection with *Flag-Fat10*, FAT10–Kv7.1 complex expression was increased in a dose-dependent manner, while the Ub–Kv7.1 complex level was unaltered in low ubiquitin expression conditions (figure 4*i*,*j*). Overall, we concluded that FAT10 competes with ubiquitin for binding to Kv7.1, thereby reducing the ubiquitination of Kv7.1 and ultimately stabilizing its expression.

### (e) FAT10 protects against the dysfunction of *I_Ks_* under hypoxia in human-induced pluripotent stem cells

**Figure 5.**
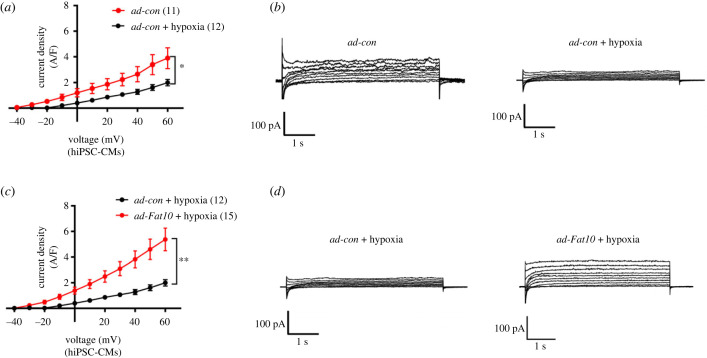
Effects of FAT10 overexpression on *I_Ks_* current densities under hypoxic stress. (*a*) Mean current density versus voltage relationships for hiPSC-CMs in the *ad-con* group (*n* = 11) and *ad-con* + hypoxia group (*n* = 12). (*b*) Representative current traces recorded in hiPSC-CMs in the *ad-con* group and *ad-con* + hypoxia group. (*c*) Mean current density versus voltage relationships for hiPSC-CMs in the *ad-con* group (*n* = 12) and *ad-Fat10* + hypoxia group (*n* = 15). (*d*) Representative current traces recorded in hiPSC-CMs in the *ad-con* + hypoxia group and *ad-Fat10* + hypoxia group. *p < 0.05, **p < 0.01, ***p < 0.001 and n.s. = not significant.

**Figure 6.**
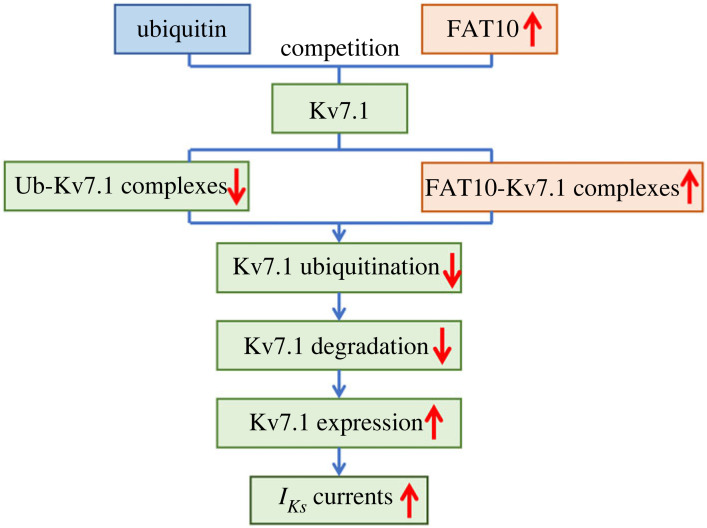
Graphical abstract by which FAT10 stabilizes Kv7.1. The graphical diagram illustrates that the overexpression of FAT10 results in a reduction in Ub–Kv7.1 complexes and an increase in FAT10–Kv7.1 complexes. Thus, FAT10 inhibits Kv7.1 ubiquitination, enhancing the expression of Kv7.1 and ultimately strengthening the *I_Ks_* currents.

Finally, we investigated the functional effect of FAT10 on *I_Ks_* under ischaemia/hypoxic conditions. To achieve this goal, we first compared the changes in *I_Ks_* currents under normal and hypoxic conditions in hiPSC-CMs. As shown in figure 5*a,b*, the current density of *I_Ks_* was significantly decreased under hypoxic stress. Then, we detected the change in *I_Ks_* currents when over-FAT10 was used under hypoxic conditions. Our results demonstrated that over-FAT10 enhanced the *I_Ks_* currents when compared with the control, which suggested that the trend of decreased *I_Ks_* currents induced by hypoxia could be alleviated through over-FAT10. Overall, the present study implied that FAT10 plays a cardioprotective role in pathological conditions (figure 6).

## 4. Discussion

The purpose of this study was to explore the effects of FAT10 on *I_Ks_* and the possible mechanism. Our study demonstrated that FAT10 competed with the ubiquitination of Kv7.1 to stabilize Kv7.1 expression, thereby enhancing the currents of *I_Ks_*. The present findings suggest a novel mechanism by which FAT10 could regulate *I_Ks_* (figure 5).

FAT10 is a relatively newly discovered UBL. Our team has previously demonstrated that FAT10 could regulate Nav1.5 by decreasing Nedd4-2/Nav1.5 complex formation, which is new evidence of the protective role of FAT10 in ischaemia-induced ventricular arrhythmia. However, little is known about whether *I_Ks_* are modulated by FAT10. Our present data showed that the currents of *I_Ks_* were enhanced when over-FAT10 was used. Consistently, our data demonstrated that the APD was significantly shorter when cells were transfected with FAT10. This study first provided novel evidence of *I_Ks_* modification by FAT10, which might be a potential target for the treatment of cardiac arrhythmias in the future.

Another interesting finding of this study demonstrated that FAT10 affects the changes in the *I_Ks_* current under ischaemia/hypoxic conditions. During cardiac ischaemia, many cardiac ion channels are susceptible to inducing cardiac arrhythmias. For example, the contribution of *I_KATP_* to repolarization was increased, while the currents of *I_Kr_* and *I_K1_* were reduced. More importantly, previous studies described that chronic ischaemia could decrease the *I_Ks_* current density and downregulate the expression of *KCNQ1* mRNA. Another study showed that Kv7.1 expression was downregulated in response to ischaemia, suggesting that the repolarization reserve of *I_Ks_* channels is important to prevent ischaemia-induced arrhythmias. Here, we found that the current density of *I_Ks_* was significantly decreased under hypoxic stress; however, the overexpression of FAT10 could ameliorate the hypoxia-induced reduction in *I_Ks_* currents. This finding adds to evidence that FAT10 protects the ischaemia heart and provides new insight for treating ischaemia arrhythmia in the future.

The UPS is an important degradation mechanism of cellular proteins including voltage-gated channels [18,19]. Ubiquitin and UBLs are a family of protein modifiers that share a certain amount of sequence and structural similarity [20]. Recently, accumulated evidence strongly supports that UBLs participate in regulating ion channels [3,10]. In the present study, we found a new regulatory mechanism by which FAT10 might regulate Kv7.1 expression but not KCNE1 to affect *I_Ks_* in ips and HEK293 cells. Therefore, the regulatory effects of FAT10 on Kv7.1 might shed light on the new role of UBLs in cardiac arrhythmias.

We investigated the specific mechanism by which FAT10 stabilizes Kv7.1 expression in NRCMs. We found that FAT10 can target Kv7.1 in cardiomyocytes through co-IP analysis. Importantly, we provided evidence that the ubiquitination level of Kv7.1 was significantly reduced under FAT10 overexpression, which was confirmed by the reverse experiment under the condition of downregulation of FAT10. In addition, our results indicate that competition between FAT10 and ubiquitin binds to Kv7.1 to form FAT10–Kv7.1 or Ub–Kv7.1 complexes. The final experiment showed evidence that FAT10 overexpression can induce the formation of the FAT10–Kv7.1 complex while reducing the level of the Ub–Kv7.1 complex. Overall, we concluded that the competitive binding of FAT10 and ubiquitin to Kv7.1 reduces the ubiquitination of Kv7.1 in cardiomyocytes thereby stabilizing the expression of Kv7.1.

In summary, we identified a novel mechanism by which FAT10 stabilizes Kv7.1 expression, by decreasing the ubiquitination of Kv7.1, thus enhancing the current of *I_Ks_*. These findings suggest that FAT10 may be a novel therapeutic target for the treatment of arrhythmia.

## Data Availability

The data are provided in the electronic supplementary material [21].
